# Effects of transcranial direct current stimulation with virtual reality on upper limb function in patients with ischemic stroke: a randomized controlled trial

**DOI:** 10.1186/s12984-020-00699-x

**Published:** 2020-06-15

**Authors:** Xiaoling Yao, Lijun Cui, Jixian Wang, Wuwei Feng, Yong Bao, Qing Xie

**Affiliations:** 1 Department of Rehabilitation Medicine, Ruijin Hospital, School of Medicine, Shanghai Jiao Tong University, Shanghai, China; 2 Deparment of Neurology, Duke University Medical Center, Durham, North Carolina USA; 3 Department of Rehabilitation Medicine, Shanghai Ruijin Rehabilitation Hospital, Shanghai, China

**Keywords:** Transcranial direct current stimulation, Virtual reality, Upper limb, Ischemic stroke, Motor function

## Abstract

**Background:**

Non-invasive brain stimulation techniques have been shown in several studies to improve the motor recovery of the affected upper-limbs in stroke patients. This study aims to investigate whether or not cathodal transcranial direct current stimulation (c-tDCS), combined with virtual reality (VR), is superior to VR alone in reducing motor impairment and improving upper limb function and quality of life in stroke patients.

**Methods:**

Forty patients who suffered ischemic stroke between 2 weeks to 12 months were recruited for this single-blind randomized control trial. The patients were randomly assigned either to an experimental group who receiving c-tDCS and VR, or a control group receiving sham stimulation and VR. The cathodal electrode was positioned over the primary motor cortex (M1) of the unaffected hemisphere. The treatment session consisted of 20 min of daily therapy, for 10 sessions over a 2-week period. The outcome measures were the Fugl-Meyer Upper Extremity (FM-UE), the Action Research Arm Test (ARAT) and the Barthel Index (BI).

**Results:**

The two groups were comparable in demographic characteristic and motor impairment. After 2 weeks of intervention, both groups demonstrated significant improvement in FM-UE, ARAT and BI scores (*P*<0.05).The experiment group demonstrated more improvement in FM-UE than the control group (10.1 vs. 6.4, *p* = 0.003) and, ARAT (7.0 vs 3.6, *p* = 0.026) and BI (12.8 vs 8.5, *p* = 0.043).

**Conclusions:**

The findings from our study support that c-tDCS, along with VR, can facilitate a stronger beneficial effect on upper limb motor impairment, function and quality of life than VR alone in patients with ischemic stroke.

**Trial registration:**

The study was registered in the Chinese Clinical Trial Registry (ChiCTR1800019386) in November 8, 2018-Retrospectively registered.

## Introduction

Motor impairment is the most common complication after stroke. Despite rehabilitation, the recovery of the affected upper limb is typically more limited than the affected lower limb [1]. There is an urgent need for emerging rehabilitation techniques to improve upper limb motor function after stroke. Recently, new therapeutic approaches including non-invasive brain stimulation, functional electrical stimulation, robotic therapies, and virtual reality (VR) have been investigated for stroke rehabilitation [2].

Transcranial direct current stimulation (tDCS) is a non-invasive brain stimulation technique which has the capability of modulating motor cortex excitability by the application of weak direct current through the scalp [3]. In general, cathodal transcranial direct current stimulation (c-tDCS) can upregulate the contralesional cortical excitability while anodal transcranial direct current stimulation (a-tDCS) can upregulate the ipsilesional cortical excitability [4–6]. Zimerman et al. Observed that the use of c-tDCS during training could increase Short Interval Intracortical Inhibition (SICI) in M1 of the contralesional hemisphere, on the contrary, decrease of SICI in M1 of the ipsilesional hemisphere. At the same time, it was observed that the c-tDCS improved the motor function of the hemiplegic hands, suggesting that there was a significant correlation between the improvement of tDCS-induced motor function and the intracortical inhibition induced by c-tDCS [7]. According to the current tDCS safety guidelines, the application of tDCS is generally safe on healthy subjects and patients with various neurological disorders including stroke [8]. tDCS has shown great promise in improving motor function during rehabilitative training of patients who have suffered from subacute ischemic stroke in several proof-of-concept stroke studies [9–11].

VR is a synthetic artificial environment which enables users to interact with multi-sensory simulation environment and receive real-time feedback [12, 13]. In general, there are two types of VR: immersion and non-immersion [12]. A study with chronic stroke patients found that after immersion VR, the activation of contralesional sensorimotor cortices (SM1s) was weakened or disappeared, with subsequently improved motor function of affected limbs [14, 15]. All these experiments indicate that VR can improve cortical plasticity and facilitate neural reorganization [16]. Similarly, another experiment performing on VR training in patients with chronic stroke also demonstrated that the functional connectivity between the contralesional motor cortex and the bilateral sensorimotor area increased significantly during finger movement. It suggested that VR training can promote brain functional connectivity and balance reconstruction between the cerebral hemispheres [17].

The learning of new motor skills depends on the feedback of the task itself in the learning process. Compared with conventional rehabilitation therapy, VR can improve the motor function of affected limbs by increasing feedback [18]. Moreover, the acquisition of motor learning or motor skills is a prerequisite for the plasticity of M1 in the motor cortex of the brain [19]. tDCS can improve the motor function of affected limbs after stroke by regulating neuroplasticity. Therefore, we hypothesize that the combination of tDCS and VR after stroke can promote the improvement of upper limb motor function to a greater extent than VR alone. We aim to test this hypothesis by conducting a single-center phase IIa study that randomized patients into tDCS+VR or VR alone.

## Methods

### Participants

Patients with ischemic stroke were recruited from the rehabilitation department of Southeast Hospital of Huangpu District, Shanghai. The Inclusion criteria are as followed: aged 18–80 years; had a first-ever ischemic stroke (silent infarct is allowed) as diagnosed by computed tomography or magnetic resonance imaging image scans; had their first ischemic stroke between 2 weeks to 12 months; can induce motor evoked potential (MEP) of contralesional first dorsal interossei muscle (FDI) using Transcranial magnetic stimulation.

The exclusion criteria were as follows: intracranial or orbital metallic implants, pacemakers or artificial cochlea; previous seizure history; previous history of brain neurosurgery or cerebral trauma; aphasia, unilateral neglect or cognitive deficits (Mini-Mental State Examination score < 20); refused to sign informed consent.

### Experimental design

This study design was a prospective, single-blinded, randomized and controlled phase II clinical trial that was conducted in Southeast Hospital of Huangpu District, Shanghai.

A rehabilitation physician who was not involved in the study used a computer to generate a random number table. The physician designated the random numbers in the random number table as the experimental group or the control group according to odd or even numbers, and the generated random distribution sequence was put into the sequentially coded, sealed and opaque envelope. When another rehabilitation physician not involved in the study was determined that the patients met the conditions, the envelope was opened in order and the patients were assigned to the corresponding group. The patients were blinded to the stimulation condition. The experimental group received combination of c-tDCS and VR therapy, and the control group received sham tDCS and VR. Both groups were simultaneously applied tDCS and VR. Each treatment programs consisted of 10 sessions (20 min/d and 5 sessions/week for 2 weeks). All patients received additional conventional occupational and physical therapies during the study period.

### Transcranial direct current stimulation (tDCS)

The electrical stimulation device was a transcranial direct current stimulation model IS300 manufactured by Sichuan Intelligent Company of China. Its two conductive rubber electrodes were placed in a saline-soaked sponge (5 × 7 cm 2) when used. The cathodal electrode was placed over the patients’ scalp which corresponded to the primary motor cortex (M1) of the unaffected hemisphere, and the region was determined by the induction of stable MEP response in the FDI using transcranial magnetic stimulation. The reference electrode was placed above the contralateral supraorbital region. The current of the experimental group was constant 2 mA for 20 min. For the control group, the current was rapidly increased to 2 mA in the beginning and then slowly tapered down to 0. At the end of the experiment, the current again rapidly ramp up to 2 mA and then slowly ramp-down to 0. It created a scalp sensation to blind the subject.

### Adverse effects’ questionnaire of tDCS

A questionnaire adapted from Poreisz et al. [8] was used to monitor common adverse effects of tDCS (e.g., tingling, itchiness, and fatigue) and its items were illustrated in Table 1. If an adverse effect was reported, the patient had to report its duration. The tDCS questionnaire, which was in the form of interview questionnaire, was administered 2 h after each tDCS session.

**Table 1:** Adverse effects’ questionnaire of tDCS

Adverse effects items	No. of patients
Tingling	4
Itching	1
Burning	0
Pain	0
Headache	0
Fatigue	0
Difficulties in concentrating	0
nausea	0
insomnia	0

### Virtual reality (VR)

The equipment for VR was made by Shanghai Fourier Intelligent Technology Co., Ltd. It consisted of a mechanical ontology, a powerful feedback sensing manipulator and a large screen providing VR scenes. The equipment was built-in with different motion mode (passive, assistant, active and resistant mode) and different game forms, mainly to help patients do exercise control training, which could effectively make the dull and monotonous rehabilitation training interesting. In this experiment, the game mode was that the patients hit the target object with the ball through the movement of the mechanical handle, but the motion mode chose depending on the capabilities of the patients. The patients were told to use only the hemiplegic hand. Meanwhile, the therapist guided and encouraged the patients to perform VR training. The duration of the VR therapy was 20 min.

### Outcome measures

The Fugl-Meyer Upper Extremity (FM-UE) [20] scale was the primary outcome to measure the motor impairment. The Action Research Arm Test (ARAT) [21] and the Barthel Index (BI) [22] were used as secondary outcome measures. ARAT was chosen to evaluate the upper limb the motor function, and BI was used to assess activities of daily living (ADL) to reflect the quality of life.

The item of the FM-UE consists of reflex, synergy, range of motion, and fine and gross hand movements. Its score ranges from 0 to 66.The reliability and validity of the scale were approved [23]. The ARAT scores are divided into grasp (6 tasks, score: 18), grip (4 tasks, score: 12), pinch (6 tasks, score: 18), and gross movement (3 tasks, score: 9) segments. The final score of ARAT is the sum of the scores from each of 4 subscales. It shows good validity [24] and sensitivity to therapy-related [25, 26] gains after stroke. The BI has 10 assessment items:bowel and bladder care, feeding, grooming, bathing, dressing, toilet use, ambulation, transfers, and stair climbing. Its total score ranges from 0 to 100.It has been shown to be a valid, responsive, and reliable measure of basic ADL in patients with stroke [27]. All evaluations were performed 1day before and after intervention by an occupational therapist who was unaware of the group assignment and study objectives.

### Statistical analysis

SPSS version 22.0 statistical software was used for statistical analysis. Descriptive statistics were performed on for all outcome variables. Independent sample t test, Chi-square test and Fisher’s exact test were used to evaluate differences in demographic and clinical characteristics between the two groups, according to whether the variables were categorical or continuous. Wilcoxon sign rank test was used to compare the data obtained before and after treatment in the two groups, due to the non-parametric distributions of the outcome measurement data. We calculated the changes of each outcome measure before and after the intervention and compared these between groups using Mann–Whitney U test. Statistical significance was set at *P* value < 0.05.

## Result

### The flow of the trial and baseline characteristics of patients

**Fig. 1:**
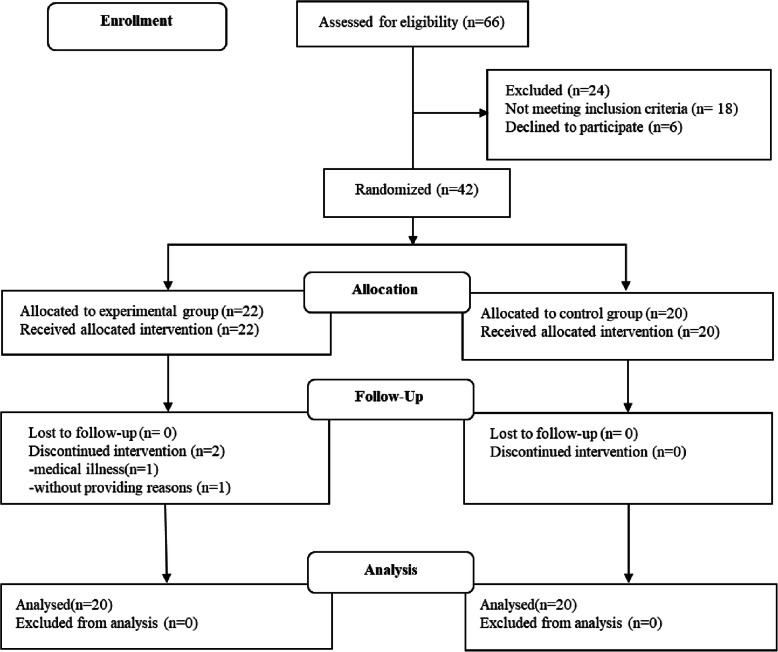
Flowchart of the trial

From April 2018 to January 2019, all inpatients in the rehabilitation department were screened. Of these, 66 patients with unilateral upper limb motor impairment due to ischemic stroke were eligible for evaluation. Among these patients, 18 patients did not meet the inclusion criteria and 6 patients declined to participate in this study.42 patients were assigned randomly to either the experimental group or the control group by using a table of random numbers.22 patients were allocated to the experimental group and 20 patients were allocated to the control group. One patient declined to continue the intervention due to medical illness and another one discontinued the intervention without providing a reason in experimental group. Therefore, a total of 40 patients (20 patients in each group) completed this study and participated in the evaluations (Fig. 1).4 patients reported tingling sensation in experimental group, lasting up to 2 min after the start of the experiment, and 1 patient reported itching sensation during cathodal stimulation in experimental group, lasting up to 1 min after the end of real stimulation (Table 1).

Table 2 shows the demographic and clinical characteristics of patients at baseline between the two groups. And there was no significant difference in age, gender, time after stroke, stroke lesion, paretic side, and stroke risk factors.

**Table 2:** Demographic and clinical characteristics of participants

Characteristic	Exp (*n* = 20)	Con (*n* = 20)	*P* value
Age (yrs)	63.0 ± 7.5	66.2 ± 6.2	0.16^a^
Gender (M/F, n)	14/6	17/3	0.45^b^
Time since stroke (d)	60.5 ± 35.5	56.5 ± 33.3	0.73^a^
Stroke lesion(n)			
cortex/subcortex/cortex and subcortex	0/15/5	1/14/5	1.00^b^
Paretic side (L/R, n)	12/8	10/10	0.53^b^
Stroke Risk Factors (n)			
Hypertension	20	18	0.49^b^
Diabetes mellitus	10	8	0.53^b^
Atrial fibrillation	2	1	1.00^b^
Hyperlipoidemia	2	1	1.00^b^

All values are mean ± SD or number. *n* number of patients, *yrs*. years, *M/F* male/female, *d* days, *L/R* left/right. ^a^*P* values by independent samples t test. ^b^*P* values by Chi-square test or Fisher’s exact test (2-tailed)

### Effects of the treatment

Table 3 shows the results of the experimental group and the control group that were recorded before and after treatment. There was no significant difference in FM-UE, ARAT, and BI at baseline between the 2 groups. After treatment, the 2 groups showed significant improvements in FM-UE, ARAT and BI by using Wilcoxon signed rank test (experimental group**:** FM-UE, ARAT, BI *P* < 0,001, respectively; control group**:** FM-UE, ARAT, BI P < 0,001, respectively).

**Table 3:** Effect of intervention on outcomes

Outcome	Exp (*n* = 20)		Con (*n* = 20)	
	Pre	Post	Pre	Post
FM-UE	24.3 ± 16.6	34.4 ± 17.8^#^	26.0 ± 15.2	32.4 ± 16.4^#^
ARAT	17.8 ± 18.4	24.8 ± 19.9^#^	15.3 ± 13.5	18.8 ± 15.9^#^
BI	59.3 ± 15.1	72.0 ± 17.1^#^	56.0 ± 11.9	64.5 ± 12.4^#^

All values are mean ± SD. *n* number of patients. ^#^*P* < 0.001 within group analysis (Wilcoxon signed rank test)

**Fig. 2:**
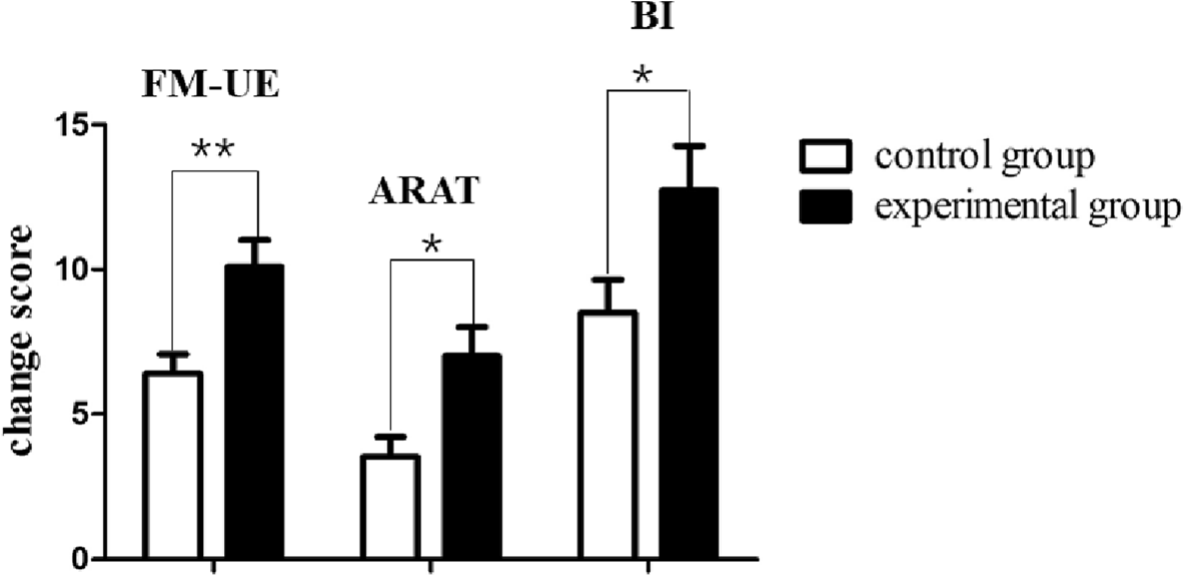
Change scores of experiment group and control group. ^**^P<0.01,^*^P<0.01: between groups change analysis

When comparing the changes of the 2 groups, greater improvements were found in the experimental group in all assessment outcomes than the control group by using Mann–Whitney U test (Fig. 2). In the post hoc analysis, the improvement of the FM-UE was statistically significant in experimental group when compared with the control group (FM-UE: 10.1 ± 4.1 in experimental group vs 6.4 ± 2.9 in control group, *P* = 0.003). The improvement of the ARAT was statistically significant in experimental group when compared with the control group (ARAT: 7.0 ± 4.5 in experimental group vs 3.6 ± 2.9 in control group, *P* = 0.026). And the improvement of the BI was also statistically significant in experimental group when compared with the control group (BI:12.8 ± 6.6 in experimental group vs 8.5 ± 5.0 in control group, *P* = 0.043).

## Discussion

Our single-center phase II study found that c-tDCS positioned over M1 of the unaffected hemisphere, combined with VR therapy for 10 sessions over a 2-week period can reduce ipsilateral upper limb motor impairment, improve motor function and enhanced ADL of patient with ischemic stroke better than VR alone. The proposed intervention is safe and tolerable as only minor adverse events were observed.

Our study is consistent with a prior study led by Lee and Chun who also investigated the effect of c-tDCS combined with VR on upper limb motor function in patients with subacute stroke. The improvement of Manual Function Test (c-tDCS+VR group VS VR group, p<0.01) and FM-UE scores(c-tDCS+VR group VS VR group, p<0.01) in the combined treatment group was significantly higher than that in the VR group, but there was no significant difference in the Korean-Modified Barthel Index scores (c-tDCS+VR group VS VR group, p>0.05) [13]. Patients included in both studies have positive motor evoked potential from the contralateral first dorsal interossei muscle, but the baseline FM-UE score of patients in their study is higher than our study (c-tDCS+VR group:38.4 ± 23.4,VR group:34.9 ± 22.0 VS our experimental group: 24.3 ± 16.6, control group:26.0 ± 15.2). They recruited patients with acute stroke (less than 1 month) while we recruit patients with a wide window (from 2 weeks to 12 months).

Viana et al. found that there was no significant difference between a-tDCS combined with VR and VR alone in FM-UE, Wolf Motor Function Test, grip strength and stroke-specific quality of life scale (SSQOL) in patients with chronic stroke(>6 months from stroke onset) [28]. The main differences between this study and our study were that the patients had a longer course of disease and a-tDCS was chosen as the target electrode in this study. Although our inclusion stated the time from stroke was 2 weeks to 12 months, but we enrolled majority of patients in the subacute phase (the average time from stroke onset is about 60 days for both groups). The brain is likely more plastible and robust for recovery in the subacute phase after stroke.

After stroke, the balance between the two cerebral hemispheres is destroyed [29, 30], and the dynamic neuroplasticity of the bilateral cerebral hemispheres is activated. However, this dynamic neuroplasticity is not always beneficial [6, 31]. tDCS induces changes in cortical excitability by regulating the conductivity of sodium and calcium channels and the activity of NMDA receptor to achieve membrane polarization [32, 33]. Pharmacological studies have shown that dextromethorphan, an antagonist of NMDA receptor, can eliminate the long-term after-effect of c-tDCS and a-tDCS [32, 33]. Bennett et al. found that NMDA receptor is involved in the regulation of cortical neuroplasticity, suggesting that tDCS may further regulate cortical neuroplasticity by regulating NMDA receptor [34]. Similar mechanisms were observed when VR improved motor function in stroke patients [14–17]. In addition to similar mechanisms, tDCS and VR may have complementary mechanisms. tDCS can improve motor skill learning by promoting BDNF secretion and TrkB activation in M1 [35]. And animal studies have shown that motor learning or acquisition of motor skills can facilitate the reorganization of the cerebral motor cortex [19, 36]. Similarly, it has been found in humans that repeated practice of the affected limb produced an effective synaptic potential to increase exercise-induced neuroplasticity [37].

The minimum clinically significant difference (MCID) of FM-UE after stroke is 4.25 points [38]. In our study, 100% of the patients in the experimental group and 65% of the patients in the control group reached this MCID, respectively. The MCID of ARAT in affected limbs after stroke is 17 points [39]. In our study, 5% of the patients in the experimental group achieved MCID, while no patients in the control group achieved MCID. This discrepancy in improvement between motor impairment and motor function is likely due to the different threshold of MCID for FM-UE (as measure of motor impairment) and ARAT (measure of motor function).

There are several limitations in this study. First, our study is single-center with a relatively small sample, the study and data need to be replicated; second, our study is a single-blind study, which may cause some bias in the results; third, as the study was conducted in the rehabilitation inpatient facility in real-world practice, and all of the patients likely received additional rehabilitation therapy outside of the clinical-trial setting. We could not quantify and control these therapies which may pose biases to the study.

## Conclusions

Our proof-of-concept single-center phase II study showed that c-tDCS combined with VR can reduce motor impairment, improve function, increase ADL in the affected upper limb in patients with subacute or chronic ischemic stroke than VR alone. This study provides critical preliminary data to plan a future multi-center clinical trial to systematically investigate the efficacy of combined intervention.

## Data Availability

The datasets used and/or analyzed during the current study are available from the corresponding author on reasonable request.

## Abbreviations

tDCS: transcranial direct current stimulation
c-tDCS: cathodal transcranial direct current stimulation
a-tDCS: anodal transcranial direct current stimulation
VR: Virtual reality
SICI: Short Interval Intracortical Inhibition
FM-UE: Fugl-Meyer Upper Extremity
ARAT: Action Research Arm Test
BI: Barthel Index
MEP: Motor evoked potential
FDI: First dorsal interossei muscle
ADL: Activities of daily living
MCID: Minimum clinically significant difference
